# Self-Extinguishing Resin Transfer Molding Composites Using Non-Fire-Retardant Epoxy Resin

**DOI:** 10.3390/ma11122554

**Published:** 2018-12-15

**Authors:** Zhi Geng, Shuaishuai Yang, Lianwang Zhang, Zhenzhen Huang, Qichao Pan, Jidi Li, Jianan Weng, Jianwen Bao, Zhengwei You, Yong He, Bo Zhu

**Affiliations:** 1 State Key Laboratory for Modification of Chemical Fibers and Polymer Materials, College of Materials Science and Engineering, Donghua University, Shanghai 201620, China; gengzhi@dhu.edu.cn (Z.G.); iamxiaopan@163.com (Q.P.); zyou@dhu.edu.cn (Z.Y.); 2 School of Materials Science and Engineering, Shanghai University, 333 Nanchen Road, Baoshan, Shanghai 200444, China; jidi123l@163.com (J.L.); wengjn@foxmail.com (J.W.); 3 SAMAC Shanghai Aircraft Manufacturing Co., Ltd., Shangfei Road, Pudong New District, Shanghai 201324, China; yangshuaishuai@comac.cc; 4 Avic Advanced Composites Center, Shijun South Street, Aviation Industrial Park, Shunyi, Beijing 101300, China; zhanglian51@163.com; 5 Collaborative Innovation Center for Civil Aviation Composites, Donghua University, Shanghai 201620, China

**Keywords:** fire retardant, tackifier, resin transfer molding

## Abstract

Introducing fire-retardant additives or building blocks into resins is a widely adopted method used for improving the fire retardancy of epoxy composites. However, the increase in viscosity and the presence of insoluble additives accompanied by resin modification remain challenges for resin transfer molding (RTM) processing. We developed a robust approach for fabricating self-extinguishing RTM composites using unmodified and flammable resins. To avoid the effects on resin fluidity and processing, we loaded the flame retardant into tackifiers instead of resins. We found that the halogen-free flame retardant, a microencapsulated red phosphorus (MRP) additive, was enriched on fabric surfaces, which endowed the composites with excellent fire retardancy. The composites showed a 79.2% increase in the limiting oxygen index, a 29.2% reduction in heat release during combustion, and could self-extinguish within two seconds after ignition. Almost no effect on the mechanical properties was observed. This approach is simple, inexpensive, and basically applicable to all resins for fabricating RTM composites. This approach adapts insoluble flame retardants to RTM processing. We envision that this approach could be extended to load other functions (radar absorbing, conductivity, etc.) into RTM composites, broadening the application of RTM processing in the field of advanced functional materials.

## 1. Introduction

Fiber-reinforced epoxy composites are widely used as structural materials to replace metal materials in airplanes, trains, and automobiles, as they possess high mechanical strength with light weight [1,2,3,4,5,6,7,8]. Resin transfer molding (RTM) is one of the most important techniques used to fabricate epoxy composites due to its cost-effectiveness, being solvent-free, the ability to be mass produced, the high automatization potential, and its excellent performance in manufacturing large parts and molding complex shapes [9,10,11,12,13,14,15].

The epoxy resin is highly flammable and needs further modification to reduce the hazard of accidental fires. Numerous efforts have focused on reducing the flammability of epoxy resins and their composites with considerable progress [16,17,18,19]. These fire-retardant epoxy composites passed the UL94 V0 flammability rating and achieved a limiting oxygen index value higher than 30% [17]. The epoxy resins used in these composites are normally prepared through introduction into resin nonflammable hardeners [20,21], building blocks for epoxy polymers [22,23], additives of small molecules or polymers [24,25,26,27], and nano/micromaterials [28,29,30]. The chemical structure, molecular weight, and composition of RTM epoxy resins were optimized to have a very low viscosity to satisfy the fluidity requirement for RTM processing, whereas most of these modification procedures increase the resin viscosity [22,29,31,32,33,34,35]. The nano/micromaterials would either be filtered by the mixer of the RTM instrument or enriched on the outer layers of reinforcing three-dimensional (3D) preforms, leading to a non-uniform distribution of the nano/micromaterials, [14,36], therefore potentially introducing a negative effect on fire resistance and the mechanical properties of the composites. 

Due to these issues, we were interested in developing an approach to manufacture self-extinguishing RTM composites without modifying the epoxy resin. Our idea was to use fire retardant-loaded tackifiers or binders to fabricate 3D glass-fiber preforms, and then the RTM epoxy composites. Tackifiers are commonly used in the RTM process to offer preforming and improved handling for the unimpregnated woven fabrics. As the tackifier resin does not soften and flow at the injection temperature, the fire-retardant tackifier layer anchored on the fabrics would not be diluted by the injected resin in the molding. This means that the composite could efficiently retard fire propagation due to the localized high content of the fire retardant. 

Red phosphorus is widely employed to enhance the fire retardancy of a variety of polymers, including epoxy resins, polyesters, and polyurethanes, due to its low cost and ecofriendly properties [37,38,39,40,41,42]. Red phosphorus is an efficient flame retardant for epoxy resins. It works in both oxygen and nitrogen-containing polymers via the formation of thermally stable char in the condensed phase and phosphorus radicals in the gas phase [43,44]. In this work, we instead used microencapsulated red phosphorus (MRP) to prepare the flame-retardant tackifier, as MRP additives are more stable and easier to handle. Using the reinforcing textile that was preformed by the modified tackifier, we prepared, by a non-fire-retardant epoxy resin and standard RTM approach, an excellent fire-retardant epoxy composite. The thermal stability and flame retardancy of the composite were both investigated by thermogravimetric analysis (TGA), the limiting oxygen index (LOI) test, the cone calorimeter test (CCT), and flammability examination. Char residues remained after the CCT, and flammability tests were observed using optical and scanning electron microscopy. To evaluate the effects of MRP loading on the mechanical properties of composites, their tensile and compression strength, modulus and interlaminar shear strength were further measured. 

## 2. Materials and Methods 

### 2.1. Materials

The epoxy resin EP3228 and tackifier powder ET3228, averaging at 10 μm, used in this study was supplied by the Avic Advanced Composites Center (Beijing, China). The MRP (HP 1250), averaging 13 μm, was purchased from the Shanghai Champ Chemical Co., Ltd. (Shanghai, China). The 2/2 twill glass-fiber fabric EW-220B-100a was purchased from the Nanjing Fiberglass Research and Design Institute Co. Ltd. (Jiangsu, China).

The modified tackifier powder was prepared by manually mixing tackifier ET3228 with MRP. MRP weight percentages in the modified tackifiers ranged from 0 to 48 wt.%. The tackifiers containing 0, 23, 38, and 48 wt.% MRP are denoted by T(0%P), T(23%P), T(38%P), and T(48%P), respectively.

### 2.2. Preforming and RTM Processing

The flame-retardant tackifier powders of 4.0 to 7.75 wt.% that were used for tackifier-fabric loading were applied uniformly on the fabrics using a sifter. The tackifier powders were melted onto the fabrics by placing fabrics under an infrared (IR) lamp at 90 °C for 10 s. Ten plies of fabrics with a 0° layup (340 mm × 70 mm) were pressed at 85 °C for 10 min by vacuum bagging to fabricate the preform.

**Scheme 1:**
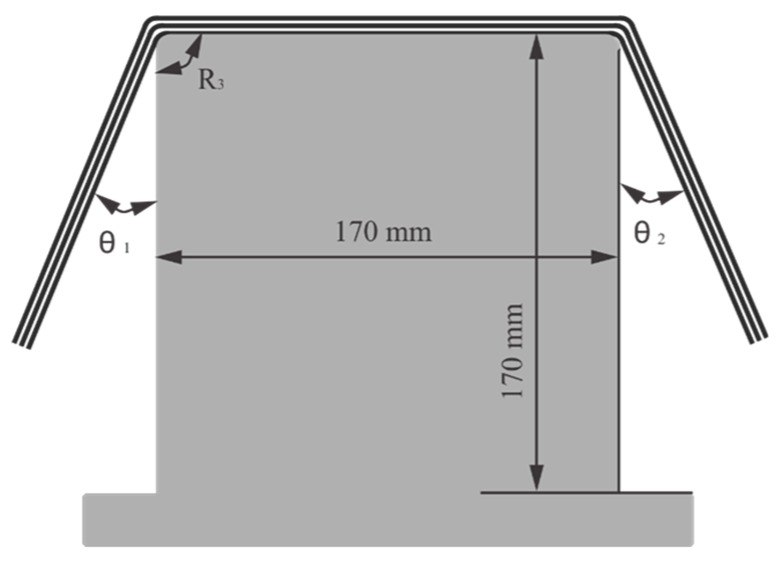
The U-shape bending test. The springback of the preform angle was determined by averaging θ_1_ and θ_2_.

U-shape bending was employed to evaluate the control of the tackifier on the preform dimensions. Ten plies of fabric with a 0° layup (340 mm × 70 mm) were fixed on a U-shape bending tool under the pressure produced by the vacuum bagging at 85 °C for 10 min. After cooling to room temperature (25 °C), the preform was removed from the tool and the springback angle was measured every 1 h (Scheme 1). The *t*-test was used to evaluate whether the differences among the springback angles of the fabrics that were preformed by tackifiers with different MRP contents were statistically significant.

**Scheme 2:**
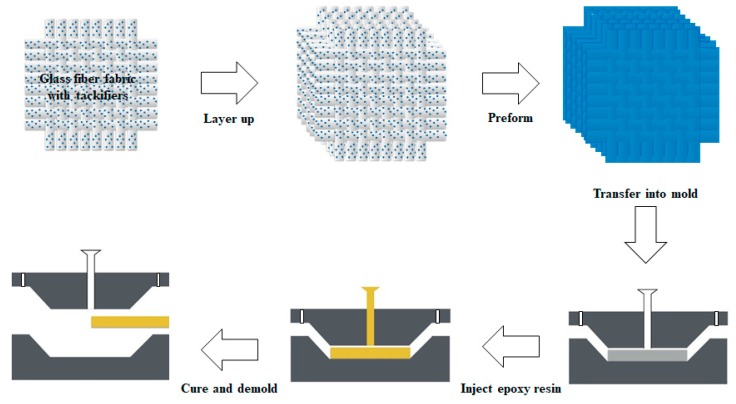
Schematic of the resin transfer molding (RTM) processing used to fabricate the fire-retardant composites using non-fire-retardant epoxy resin.

The standard RTM approach was carried out to fabricate glass-fiber-reinforced epoxy resin composites. The preform containing 10 plies of fabrics was placed into a rectangular RTM mold with injection and outlet ports. The assembled mold was heated to 50 °C while a vacuum was applied to the mold cavity. Epoxy resin was injected into the mold at a pressure in the range of 0.05 to 0.35 MPa while the thermostat was set at 50 °C. After resin injection, the mold temperature was maintained at 100 °C for 1 h to cure the composite. A post-cure process was performed by maintaining the temperature at 140 °C for 2 h. After the assembled mold was cooled to room temperature, the composite was demolded (Scheme 2). 

The composites that were preformed by the tackifiers containing 0, 23, 38, and 48 wt.% MRP are denoted as COMP-T(0%P), COMP-T(23%P), COMP-T(38%P), and COMP-T(48%P), respectively. The cured resin samples containing 0, 2.5, 5, and 7.5 wt.% MRP are denoted as Resin(0%P), Resin(2.5%P), Resin(5%P), and Resin(7.5%P), respectively.

### 2.3. Rheological Measurement

Dynamic rheological measurements of resins were performed with a dynamic oscillatory rheometer (AR2000, TA Instrument, New Castle, DE, USA). For each measurement, the sample was heated from 80 to 110 °C at a heating rate of 2 °C/min.

### 2.4. Thermal Analysis

Thermogravimetric (TG) experiments were performed using an analyzer (STA 409PC, Netzsch Group, Bavaria, Germany). Eight milligram samples were heated in alumina pans from room temperature to 800 °C at a heating rate of 10 °C/min in air. To evaluate the effect of MRP loading on the thermal stability of ET3228, a simple sum of char residuals of MRP and ET3228 (m_sum_) was calculated on the basis of the Equation (1). The char residuals of ET3228 and MRP at 600 °C are m_t_ and m_p_, respectively, and c_t_ and c_p_ are the weight percentages of ET3228 and MRP in the flame-retardant tackifier, respectively.

m_sum_ = m_t_ × c_t_ + m_p_ × c_p_ (1)

### 2.5. Morphological Characterization

Optical microscopy (BX51 M, Olympus, Tokyo, Japan) was performed to observe the distribution of the ET3228/MRP tackifier in the molded composites. The cross-sections of the samples were wet-polished to prepare smooth surfaces for microscopy observation. Scanning electron microscopy (SEM; S-4800, Hitachi, Hitachi, Japan) was carried out to examine the sample morphology before and after combustion.

### 2.6. Fire-Retardancy Evaluation

A butane torch (BS-271, Wenzhou Wans Torch Co. Ltd., Wenzhou, China) was used to ignite the composite samples for 10 s; then, we measured the burning time *t*_1_. If the flame died, we reignited it for 10 s more and again measured the secondary burning time *t*_2_. The results of the flammability examinations are shown in Video S1 in the Supplementary Material.

The limiting oxygen index (LOI) was measured on an oxygen index meter (PX-01-005, Suzhou Phinix Instrument Co., Ltd., Suzhou, China) according to ISO 4589-2:1996. The size of the specimens used in the test was 150 mm × 10 mm × 4 mm. The *t*-test was used to evaluate whether the differences among the LOI values of the resins or composites with different MRP contents was statistically significant.

The cone calorimeter test was performed on a Fire Testing Technology cone calorimeter (Stanton Redcroft, Fire Testing Technology Ltd., East Grinstead, UK) according to ISO 5660-1:2015 [45]. Each sample (with a size of 100 mm × 100 mm × 3 mm) was placed on an aluminum foil and exposed horizontally to an external heat flux of 50 kW/m^2^. 

### 2.7. Mechanical Properties

The mechanical properties were determined on a fatigue-testing system (8803, Instron, Norwood, MA, USA). The tensile, compression, and interlaminar shear strengths and modulus were each tested according to ASTMD3093 [46], ASTMD6641 [47], and ASTMD2344 [48], respectively. The *t*-test was used to evaluate whether the differences among the strengths and moduli of the composites with different MRP contents were statistically significant.

## 3. Results and Discussion

### 3.1. Preforms by Flame-Retardant-Loaded Tackifier 

We prepared the fire-retardant ET3228 tackifiers with MRP weight contents of 23, 38, and 48 wt.% and included pristine ET3228 as a control. The main reason for using the ET3228 tackifier to stabilize the preform dimension was that its viscosity is much higher than that of the EP3228 resin at the injection temperature of 50 °C. To clarify the effect of MRP addition on tackifier viscosities, we registered the temperature dependences of complex viscosity at 1 Hz for these flame-retardant-loaded tackifiers. We found that the addition of MRP enhanced the viscosity of tackifier resins. 

**Figure 1:**
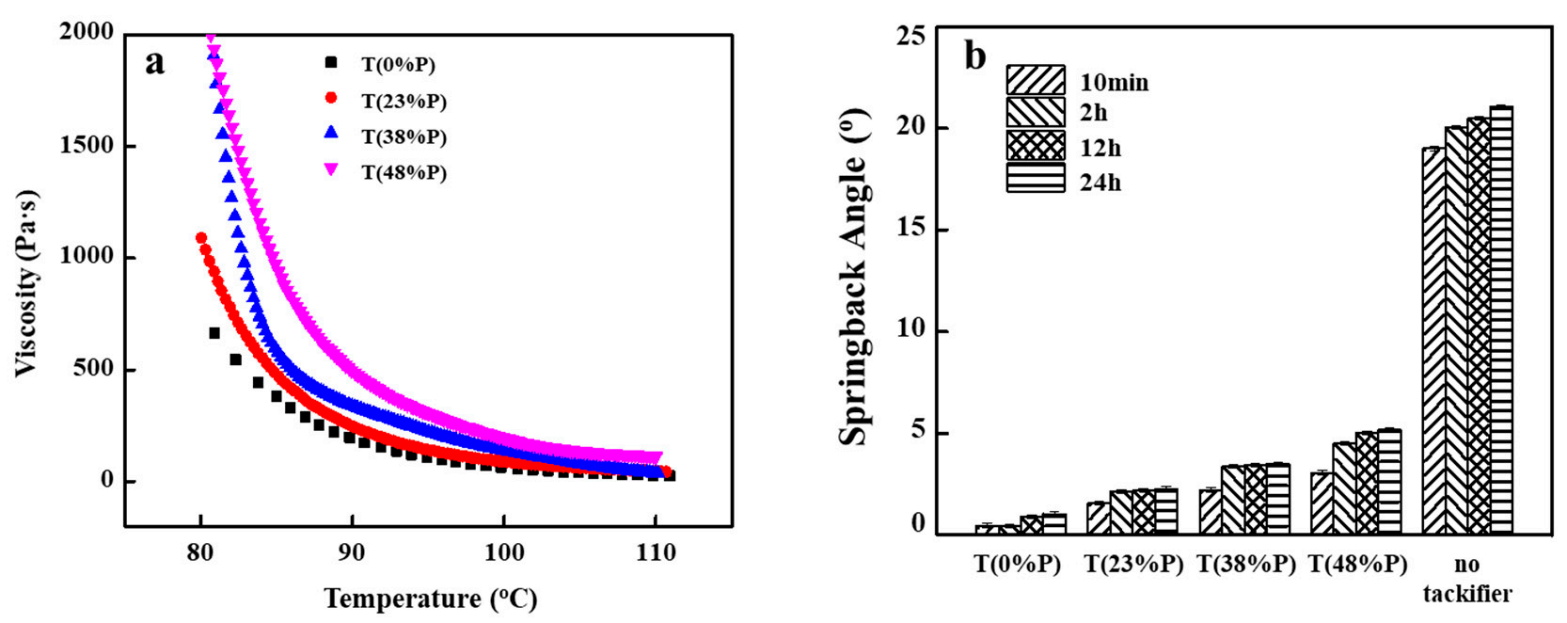
(**a**) Temperature dependence of complex viscosities for the ET3228 tackifiers with microencapsulated red phosphorus (MRP) contents ranging from 0 to 48 wt.%. (**b**) Springback angles monitored for the fabrics preformed by 0–48 wt.% MRP-loaded ET3228 tackifiers and the fabric without preforming. All the *t*-tests carried out on the springback angles of the fabrics preformed by different tackifiers resulted in a *p* value less than 0.01, indicating that the dependence of the springback angle on MRP content is statistically significant.

As noted, with the MRP content increasing from 0 to 48 wt.%, the complex viscosity of the tackifier at 85 °C increased from 381 to 940 Pa·s (Figure 1a). The increase in viscosity normally restrains tackifier resins from wetting fabrics and reduces their control over the preform dimension [49]. We employed the U-shape bending test to evaluate the effect of MRP addition on its control over the preform dimension [49]. When we prepared the preforms, the total amount of the tackifier consisting of the ET3228 resin and MRP varied from 4.0 to 7.75 wt.% of the preform, whereas the amount of the ET3228 resin was fixed at 4.0 wt.%. Ten plies of fabric were molded on an 85 °C U-shape bending tool, where the pressure was produced through vacuum bagging for 10 min (Scheme 1). After cooling to room temperature (25 °C), the preform was removed from the tool and the springback angle was monitored over time. The springback angles for the tackifiers with 0, 23, 38, and 48 wt.% MRP additives were found to be 1.0, 2.3, 3.5, and 5.2°, respectively (Figure 1b). Compared to the springback angle for the preform without using a tackifier (21.1°), the springback angles for the modified tackifiers were much smaller. Based on previous studies and our experiences, a preform with a springback angle smaller than 6° is acceptable, especially when the fiber volume faction is controlled to be lower than 55 vol.% [50]. The preforming control was improved by extending the duration of preforming or processing at a temperature higher than 85 °C. As such, MRP-loaded tackifiers more thoroughly impregnated the fabrics [9,50]. However, to fairly evaluate the effects of MRP addition on the composite properties, we did not change any condition or parameter of RTM processing in this study.

**Figure 2:**
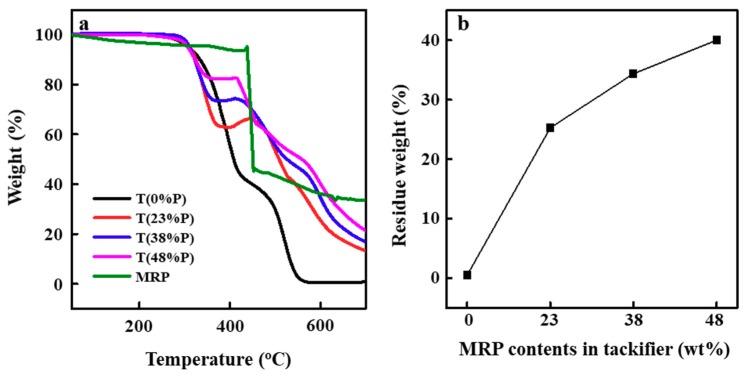
(**a**) Thermogravimetric registrations and (**b**) residues when heated to 600 °C under an air atmosphere for ET3228 tackifiers with MRP contents in the range of 0 to 48 wt.%.

We used TG experiments to evaluate the effects of MRP addition on the thermal stability of ET3228 tackifiers in air. The results of thermogravimetric analysis (TGA) and the char residual at 600 °C are shown in Figure 2a,b, respectively, and the first derivative TGA results (DTG) are shown in Figure S1. The ET3228 tackifier without MRP presents a two-step decomposition with two maximum mass-loss rates at 395 and 525 °C. The first step is the fast thermal decomposition of the ET3228 tackifier and the second step is further oxidative degradation of char residues [51]. The tackifier without MRP did not leave any residue at 600 °C. MRP decomposed in a single step with a maximum loss rate at 445 °C and left a residue of 34.6 wt.%. By mixing the ET3228 tackifier with MRP, its decomposition behaviors occurred in three steps, with maximum mass-loss rates at around 330, 475 and 600 °C. This indicates that the MRP loading promotes the thermal decomposition of the tackifier but distinctively depresses the oxidative degradation of char residues. In addition, an increase in weight was observed around 450 °C for most MRP-loaded tackifiers, which could be attributed to the oxidation of phosphorus in the condensed phase [52].

By comparing the decomposition behaviors and residues at 600 °C, we found that the addition of MRP dramatically enhanced thermal stability under an air atmosphere. With increasing MRP content from 0 to 48 wt.%, the residues of the flame-retardant tackifiers increased sharply at 600 °C. Notably, their residues were much more than the simple sum of the residues of the two individual materials. For example, the residue for the tackifier with 48 wt.% MRP was 40.0%, whereas the simple sum of the residues of the two individual materials was calculated to be 17.3 wt.%. The details of this calculation were described in the Materials and Methods section. This phenomenon is attributed to the enhanced formation of carbonaceous layers containing a large amount of stable P–O and P–C complexes [53]. The char residue inhibited oxygen penetration and heat conduction and retarded the thermal decomposition process.

### 3.2. Self-Extinguishing RTM Composites with Flammable Resins

The MRP weight percentages in the resins were 0, 2.5, 5, and 7.5 wt.% for the composites preformed by the tackifiers, with MRP contents of 0, 23, 38, and 48 wt.%, respectively. The MRP and tackifier contents in the composites and preforms are shown in Tables S1 and S2, respectively. The design of the flame-retardant tackifier possessed significantly beneficial properties for fabricating flame-retardant composites with typical RTM processing conditions. Due to the MRP being loaded in the tackifiers, the viscosity of the EP3228 resin could not be influenced by the flame-retardant MRP, which ensures the high resin fluidity necessary for wetting fabrics and driving air bubbles at 50 °C. 

To evaluate the effects of MRP addition on resin viscosity, we directly mixed the MRP into the EP3228 resin in similar contents and tested the resin viscosity. As shown in Figure S2, the viscosity of the EP3228 resin increased from 0.35 (0 wt.% MRP) to 0.63 (7.5 wt.% MRP), corresponding to an 80% viscosity increase. The 80% increased viscosity for the MRP-loaded EP3228 resin weakened its fluidity, which made it difficult to mold the composites following the standard RTM approach for EP3228 resin. Adopting a higher injection pressure and temperature may help resins to wet fabrics and improve the composite quality. However, we found the 13 μm MRP particles would be trapped in the outer fabric layer of the preforms as the gap between fibers of fabrics is around 2 μm. Even for nanoparticles, previous works have revealed their non-uniform distribution and uneven performance [14,36]. These issues normally restrain the use of non-soluble additive modified resins in RTM processing. 

**Figure 3:**
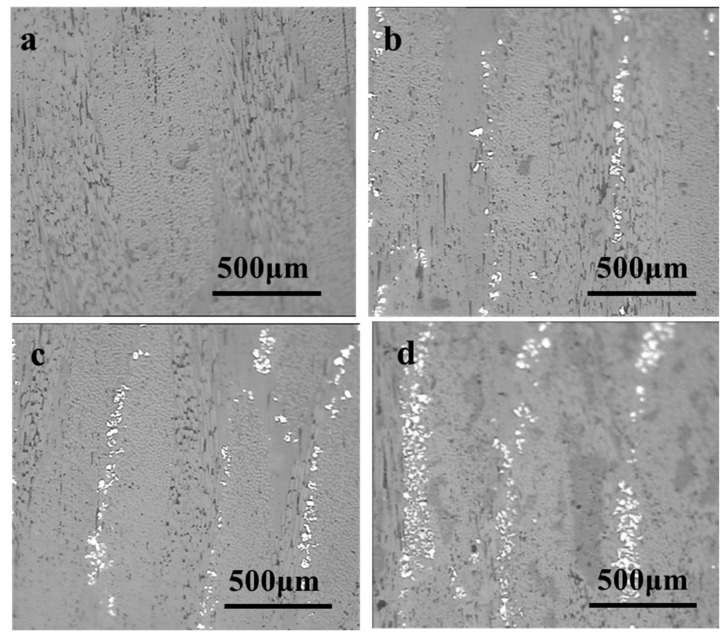
Spatial distribution of MRP in resin transfer molded (RTM) composites. Optical microscope images for the sections of RTM epoxy composites preformed by ET3228 tackifiers containing (**a**) 0, (**b**) 23, (**c**) 38 and (**d**) 48 wt.% MRP. The sections of the composites are vertical to the reinforced fabrics.

Employing flame-retardant tackifiers leads to MRP concentration on the fabric surface. We used an optical microscope to observe the composite section (Figure 3). MRP additives were enriched on the surface without penetrating the fabric. This was a reasonable finding as the tackifier resin did not soften at the injection temperature and the MRP additives were anchored on the fabric surface during resin injection. The unique MRP distribution, i.e., the enrichment of MRP particles on the fabric surface, should be responsible for the composites’ excellent fire retardancy, which is demonstrated below. 

**Figure 4:**
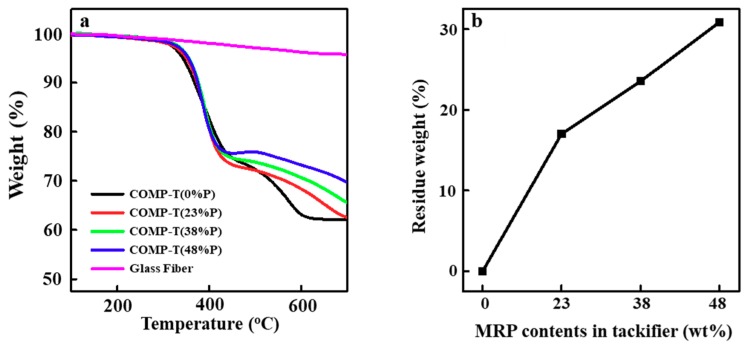
(**a**) Thermogravimetric registrations with a heating rate of 10 °C min^−1^ under an air atmosphere and (**b**) calculated residues (600 °C) of the organic material, including the resin, tackifier, and fiber sizing coating, for the RTM epoxy composites preformed by ET3228 tackifiers with MRP content in the range of 0 to 48 wt.%.

We used thermogravimetric measurements to investigate the effect of MRP addition on the thermal decomposition of composites. As shown in Figure 4a, the weight of the composite without MRP loading in the tackifier reached a constant when the temperature increased above 600 °C. This indicates that all the organic materials were gasified and that the remaining 63.2 wt.% residue originated from the glass fabric [54]. Thus, the weight percentage of the organic materials was 36.8 wt.% for the composite. Notably, the organic materials consisted not only of the resin and tackifier, but also the fiber sizing coating, the presence of which was proven by its 3.8 wt.% weight loss at 600 °C. To better understand the effect of MRP addition on the decomposition behavior, we calculated the residues relative to the organic materials at 600 °C for all composites by dividing the difference between the composite residue with MRP and that of the composite without MRP with the weight percentage of the organic materials (36.8 wt%), which is shown in Figure 4b. In this case, the density difference between the MRP additive and the tackifier were not considered. With the addition of MRP, their residues relative to the organic materials increased sharply. As noted, the residue reached 30.9 wt.% when the tackifier was loaded with 48 wt.% MRP (corresponding to 7.5 wt.% of the whole resin). The 30.9 wt.% residue was much more than the residue that the MRP additive produced by itself, as it only accounted for 7.5 wt.% of the resin. Thus, this result provides evidence for the enhanced thermal stability of the composites. 

**Figure 5:**
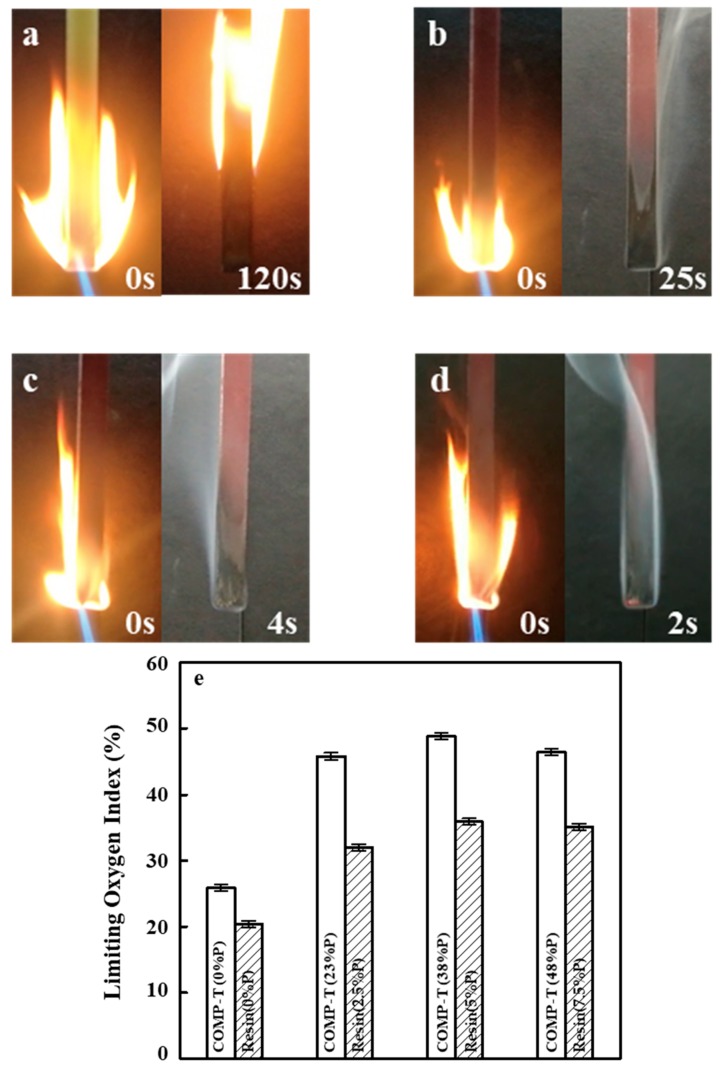
Digital pictures taken during the first burning of RTM epoxy composites preformed by ET3228 tackifiers containing (**a**) 0, (**b**) 23, (**c**) 38, and (**d**) 48 wt.% MRP, where the time was counted after the sample had been ignited for 10 s. (**e**) Limiting oxygen index (LOI) values for the RTM composite samples preformed by the ET3228 tackifiers containing 0, 23, 38, and 48 wt.% MRP (white columns), and the cured EP3228 resin samples containing 0, 2.5, 5, and 7.5 wt.% MRP (gray columns). All the *t*-tests carried out on the LOI values of the composites and cured resins resulted in a *p* value less than 0.001, indicating that the dependences of LOI value on MRP content for the composites and cured resins are statistically significant.

To evaluate the fire retardancy of the composites in the lab, we directly examined the flammability of the composite samples [55,56]. The results are summarized in Figure 5a–d and Table S3. The composite sample without MRP was easily ignited and kept burning until all the organic materials were consumed. However, when 23 wt.% MRP (corresponding to 2.5 wt.% of the whole resin) was added into the tackifier, the composite self-extinguished within 25 s (t_1_). When the MRP content in the tackifier was higher than 38 wt.% (corresponding to 5 wt.% of the whole resin), the first burning time was dramatically reduced to less than 4 s, indicating excellent fire retardancy. By increasing the MRP weight content to 48 wt.%, the first burning time was further reduced to 2 s. We also noted that the secondary burning behaviors of the composites were very similar to the first behaviors presented above. 

To accurately define the composites’ fire retardancy, we used the limiting oxygen index (LOI) measurement and cone calorimeter test to clarify the flammability of composites. The LOI is defined as the minimum fraction of oxygen in an oxygen nitrogen mixture to sustain combustion after ignition. The LOI value of the composite without MRP was about 25.9% (Figure 5e). By introducing 23 wt.% MRP (corresponding to 2.5 wt.% of the whole resin) into the tackifier, the LOI value of composite increased to 46.0%. By increasing the MRP content to 38 wt.% (corresponding to 5.0 wt.% of the whole resin) in the tackifier, the LOI reached a maximum value at 48.8%, which meant an 88.42% increase in fire retardancy. We noted that any further increase in MRP content did not increase the LOI value any more. The LOI value for the composite preformed by the tackifier containing 48 wt.% MRP became smaller (46.4%). For the cured EP3228 resins mixed with MRP, their LOI values increased from 20.4 (0 wt.%) to 35.1 (7.5 wt.%), which is also shown in Figure 5e. However, it was difficult to evaluate the effect of MRP distribution on fire performance by comparing the two samples, as the fabrics also contribute to the fire retardance of composites.

**Figure 6:**
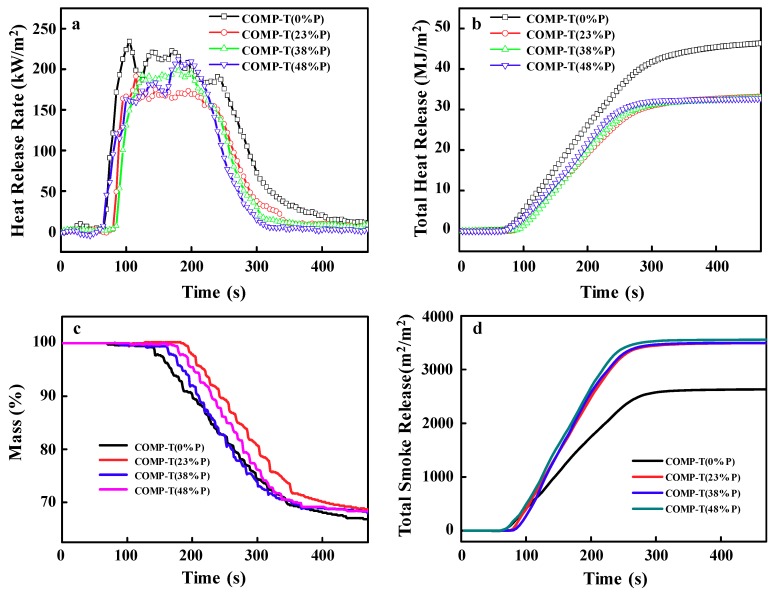
(**a**) Heat release rate, (**b**) total heat release, (**c**) mass loss, and (**d**) total smoke release for the RTM epoxy composites preformed by the ET3228 tackifiers with a MRP content in the range of 0 to 48 wt.%.

The cone calorimetry tests are widely considered to be one of the most critical bench-scale measurements in the field of fire testing. At a constant heat flux of 50 kW/m^2^, we recorded the heat-release rate curves, weight loss curves and total smoke release (TSR) to evaluate the composite flammability (Figure 6). The main cone calorimeter testing data are shown in Table S4. As shown in Figure 6a, the heat release rate for the composite was distinctively reduced by adding only 23 wt.% MRP (corresponding to 2.5 wt.% of the whole resin) into the tackifier. By further increasing the MRP content in the tackifier, the heat release rate tended to be slightly faster, but still slower than the composite without MRP. To understand the total heat release of the composites, we integrated the heat-release rate curve and plotted the results in Figure 6b. We noted that the addition of 23 wt.% MRP (2.5 wt.% of the whole resin) into the tackifier markedly decreased the total heat release from 46.12 to 33.02 MJ/kg. This corresponded to a 28.4% reduction in the total heat release. Any further increase in MRP contents did not noticeably reduce the total heat release. The total heat release for the composite preformed by the tackifier containing 48 wt.% MRP was 32.64 MJ/kg, which indicated a 29.2% reduction in the total heat release. Figure 6c presents the mass-loss curves of these composites measured by the CCT. The composites preformed by the ET3228 tackifiers containing 23 wt.% MRP started to decompose at 180 s, while the neat composite started at 135 s. This indicates the enhanced thermal stability of the former. By increasing the amount of MRP in the tackifiers, the decomposition of composites started earlier, but was still much slower than in the neat composite. All the MRP-loaded composites produced more residue than the neat sample. Figure 6d illustrates the TSR of the composites during the CCT. The TSR showed a 33% increase after adding 23 wt.% MRP in the tackifier when compared to the neat epoxy composite. However, the TSR did not noticeably increase for composites with higher MRP contents in the tackifier.

**Figure 7:**
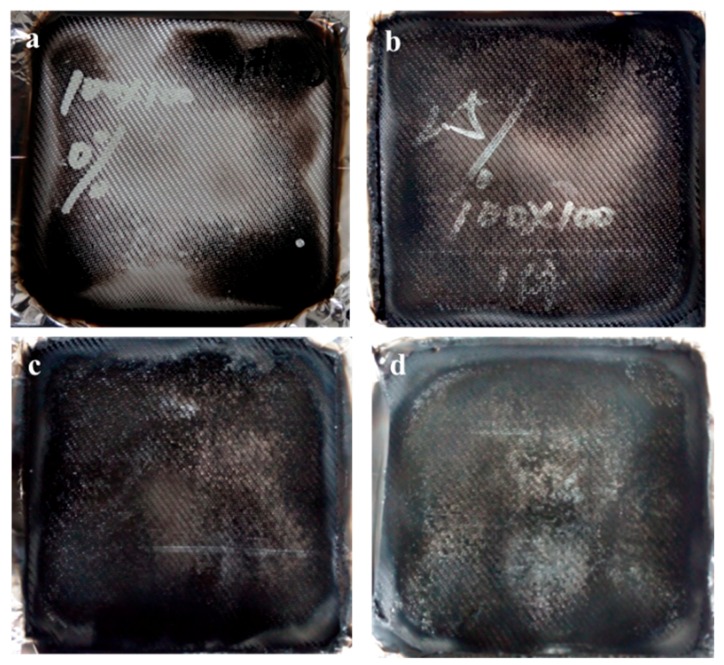
Digital pictures for RTM epoxy composites preformed by ET3228 tackifiers containing (**a**) 0, (**b**) 23, (**c**) 38 and (**d**) 48 wt.% MRP after the cone calorimeter test.

**Figure 8:**
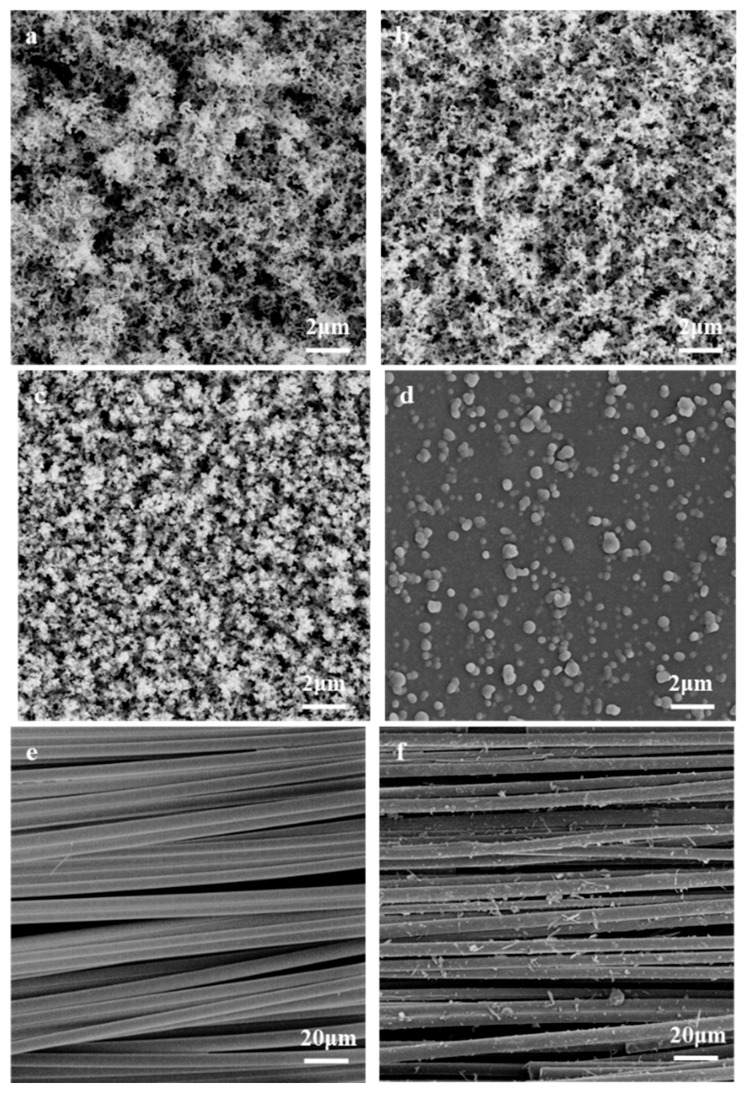

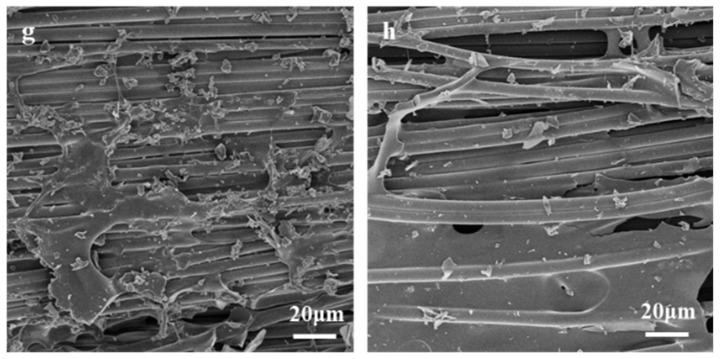
Scanning electron microscopy (SEM) images of the external surfaces of burned RTM epoxy composites preformed by ET3228 tackifiers containing (**a**) 0, (**b**) 23, (**c**) 38 and (**d**) 48 wt.% MRP, and inner surfaces of burned RTM epoxy composites preformed by ET3228 tackifiers containing (**e**) 0, (**f**) 23, (**g**) 38 and (**h**) 48 wt.% MRP.

The reduction in heat release could be attributed to the enhanced char formation with the addition of MRP, which was obvious in the digital images of the composites after the cone calorimetry tests (Figure 7). The char residue microstructures for the composites after the burning test were also observed by SEM. As shown in Figure 8a–d, more compressed and continuous char residues were formed on the external composite surface with high MRP contents. Figure 8e–h illustrates the morphologies of the composite inner layers after the burning test. The tackifier and resin around the glass-fiber fabrics were completely burned in the composite without MRP. In the composites containing MRP, residue formation was clearly observed inside the composites, and the residue amount correlated well with the MRP content in the tackifiers. The SEM images for the external and fracture surfaces of the composites before flaming are shown in Figure S3 for comparison.

We considered attributing this dramatically enhanced fire retardancy to the unique heterogenous distribution of MRP additives in the composites. The MRP additives were enriched on the fabric surface without being diluted by the injected resin because the tackifier did not soften during resin injection. MRP enrichment promoted the formation of concentrated char residues on the fabric surface [57]. The thick char layer on the fabrics could act as a barrier to effectively retard heat-flux and flammable–volatile permeation, suppressing burning propagation. We noted that the MRP additives could not penetrate and distribute into the resins inside fabrics (Figure 3), which prevents promoting the charring of resins inside fabrics. Due to the size of the MRP particles being much larger than the gap between the fibers, the MRP particles could not penetrate inside the fabrics and the optimal conditions could not be reached for further improving the flame retardancy of the composites. However, further work combining the fire-retardant epoxy resin with our MRP modified preforms is expected to produce a much better fire-retardant composite.

### 3.3. Composite Mechanical Properties

**Figure 9:**
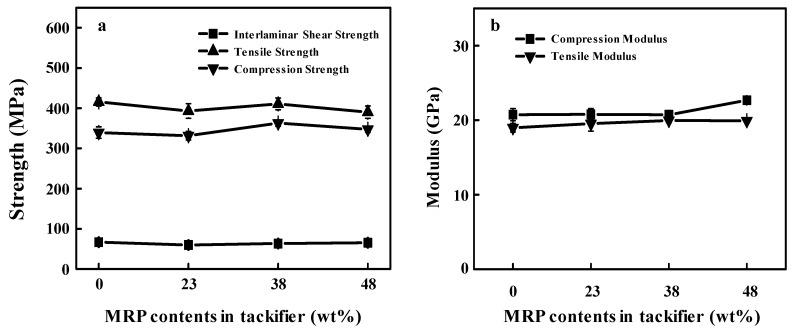
Mechanical properties of RTM epoxy composites. (**a**) Interlaminar shear, tensile, and compression strength values of RTM epoxy composites preformed by ET3228 tackifiers with an MRP content in the range of 0 to 48 wt.%. (**b**) Tensile and compression modulus values of RTM epoxy composites preformed by ET3228 tackifiers with an MRP content in the range of 0 to 48 wt.%. All the *t*-tests on the interlaminar shear, tensile, and compression strengths of composites resulted in a *p* value less than 0.05, indicating that the dependences of interlaminar shear, tensile and compression strength on MRP content are statistically significant. The *t*-test on the moduli of composites resulted in a *p* value larger than 0.05, indicating that the dependences of tensile and compression moduli on MRP content are not statistically significant.

We successfully demonstrated that the application of a flame-retardant tackifier significantly enhanced the flame resistance of RTM composites. The unique beneficial aspect to this approach is that when molding fire-retardant composites by RTM, we did not need to modify the resins. However, this technique can potentially reduce the mechanical strength of the composites, as it introduces MRP additives to the fabric-resin interface. With respect to this issue, we carried out a systematic study on the mechanical properties of the composites. The interlamellar shear strength slightly decreased when 23 wt.% MRP was added in the tackifier but recovered with further loading of MRP additives (Figure 9a). This indicates that the interlamellar shear strength is not sensitive to MRP loading in tackifier resin. We observed a slight fluctuation (either an increase or decrease) in the tensile strength and a very slight increase in the compression strength for the composite preformed by tackifiers with MRP (Figure 9a). Thus, these data do not support the notion that the effect of the MRP addition on the mechanical strengths of composites was distinctive. In comparison, the effect on the tensile and compression moduli was clear and regular, although the moduli increase with MRP loading was also slight (Figure 9b). We noted that without loading MRP, the interlamellar shear strength (ILSS, 66.8 MPa) was much higher than those (30–40 MPa) reported previously [58,59], and the compression and tensile strengths were comparable to, or even higher than those reported in previous works [60,61,62].

Several reasons explain the indistinctive effects of MRP loading on the mechanical properties. First of all, the small size (13 μm) of the MRP additives used here would reduce the effect that adding MRP would have on mechanical properties [38]. Previous studies found that the mechanical properties are sensitive to the size of MRP additives. When the size of MRP additives is smaller than 16 μm, the damage caused by the addition of MRP to the mechanical properties can be greatly reduced [38]. In addition, the MRP additives used in this work could have formed a strong interfacial interaction between the encapsulating layer and the resins, which benefited MRP dispersion and the retention of any mechanical properties [38]. Finally, increasing viscosity in the tackifiers with the addition of MRP could also help improve the composites’ mechanical properties. As we discussed above, the viscosity increase in the tackifiers would restrain the tackifier resins from wetting the fibers inside the fabric tows. This would also reduce tackifier control over the preform dimension and leave more tackifiers outside the fiber tows. However, in this case, the void content would decrease and the composite mechanical properties would instead improve [49]

## 4. Conclusions

The introduction of fire-retardant additives or building blocks into resins is generally adopted to improve the fire retardancy of resins. However, the concomitant increase in resin viscosity and the enrichment of insoluble additives on the preform outer layers remain challenges for RTM processing. Targeting this issue, we developed a robust approach to fabricate self-extinguishing RTM composites using non-fire-retardant epoxy resins. We loaded the halogen-free flame retardant, microencapsulated red phosphorus (MRP) into tackifiers instead of resins. The flame-retardant-loaded tackifiers retained the preform shapes and integrity of the preforms during resin injection. The flame-retardant enrichment on fabric surfaces, due to the high viscosity of tackifiers, endowed the composites with excellent fire retardancy while causing almost no effect on the mechanical properties. After loading 48 wt.% MRP in the tackifier (accounting for 5 wt.% of the whole resin), the flammable composite became self-extinguishing within two seconds while retaining a springback angle of 5.2°. Its LOI index was enhanced by 79.2%, and its total heat release was reduced by 29.2%. As this approach does not modify the resins, the RTM processing does not need to be reoptimized. One of most attractive properties of this approach is that it makes the insoluble flame retardant adaptable to RTM processing. This approach is basically applicable to all RTM resins for fabricating composites if an appropriate tackifier is available. This approach is not limited to fire-retardant loading, but could be extended to load other functions, such as radar absorbing, conductivity, etc., into RTM composites.

## Supplementary Materials

The following are available online at http://www.mdpi.com/1996-1944/11/12/2554/s1: Figure S1: Derivative thermogravimetric analysis (DTG) for MRP and ET3228 tackifiers with microencapsulated red phosphorus (MRP) contents in the range of 0–48 wt.%, Figure S2: Temperature dependence of complex viscosities for the EP3228 resin mixing with MRP content in the range of 0–7.5 wt.%, Figure S3: Scanning electron microscopy (SEM) images of the external surfaces of resin transfer molded (RTM) epoxy composites preformed by the ET3228 tackifiers containing (**a**) 0, (**b**) 23, (**c**) 38, and (**d**) 48 wt.% MRP, and fracture surfaces of RTM epoxy composites preformed by the ET3228 tackifiers containing (**e**) 0, (**f**) 23, (**g**) 38, and (**h**) 48 wt.% MRP before flaming, Table S1: MRP and ET3228 contents in composites, Table S2: MRP and ET3228 contents in preforms, Table S3: First burning time (t_1_), secondary burning time (t_2_), and dripping observed for RTM composite samples preformed by the ET3228 tackifiers containing 0, 23, 38, and 48 wt.% MRP, and cured resin samples containing 0, 2.5, 5, and 7.5 wt.% MRP, Table S4: Combustion calorimetric test (CCT) results for the composites. Video S1: Flammability examination.
