# Supplementary material for: Self-Extinguishing Resin Transfer Molding Composites Using Non-Fire-Retardant Epoxy Resin
Source: Materials (Basel). 2018 Dec 15;11(12):2554. doi: 10.3390/ma11122554 (PMC6315867; doi:10.3390/ma11122554)
Supplement: Supplementary file 1 [file materials-11-02554-s001.zip › materials-376173-SI.pdf]

Supporting Information for:

Self-Extinguishing Resin Transfer Molding Composites Using Non-Fire-Retardant Epoxy Resin

Zhi Geng[1,2,∇], Shuaishuai Yang[3,∇], Lianwang Zhang[4], Zhenzhen Huang[2,*] , Qichao Pan[1,2], Jidi Li[2], Jianan Weng[2], Jianwen Bao[4,*] , Zhengwei You[1], Yong He[5,*] and Bo Zhu[2,*]

[1]State Key Laboratory for Modification of Chemical Fibers and Polymer Materials, College of Materials Science and Engineering, Donghua University, Shanghai 201620, China; gengzhi@dhu.edu.cn (Z. G.); Hzzdhu@gmail.com (Z. H.); iamxiaopan@163.com (Q. P.); zyou@dhu.edu.cn (Z. Y.).

[2]College of Materials Science and Engineering, Shanghai University, 333 Nanchen Road, Baoshan, Shanghai 200444, China; gengzhi@dhu.edu.cn (Z. G.); iamxiaopan@163.com (Q. P.); jidi123l@163.com (J. L.); wengjn@foxmail.com (J. W.); bozhu@shu.edu.cn (B. Z.).

[3]SAMAC Shanghai Aircraft Manufacturing Co., Ltd, Shangfei Road, Pudong New District, Shanghai 201324, China; yangshuaishuai@comac.cc (S. Y.).

[4]Avic Advanced Composites Center, Shijun South Street, Aviation Industrial Park, Shunyi, Beijing 101300, China; zhanglian51@163.com (L. Z.); baojw@avic.com (J. B.).

[5]Collaborative Innovation Center for Civil Aviation Composites, Donghua University, Shanghai 201620, China; yhe@dhu.edu.cn (Y. H.).

[∇]These authors contributed equally to this work.

*Correspondence: bozhu@shu.edu.cn (B. Z.); hwangzz@163.com (Z. H.); baojw@avic.com (J. B.); yhe@dhu.edu.cn (Y. H.)

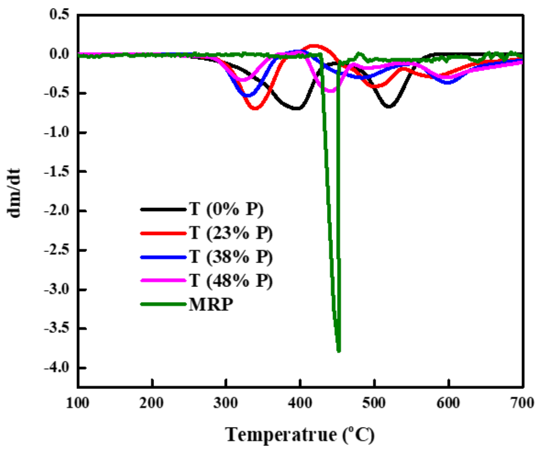

Figure S1. Derivative thermogravimetric analysis (DTG) for MRP and ET3228 tackifiers with MRP contents in the range of 0-48 wt%.

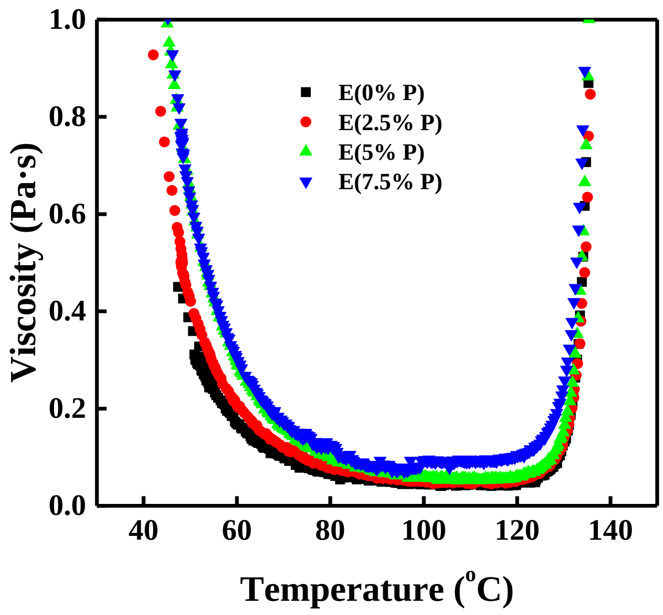

Figure S2. Temperature dependence of complex viscosities for the EP3228 epoxy resin mixing with MRP content in the range of 0–7.5 wt%.

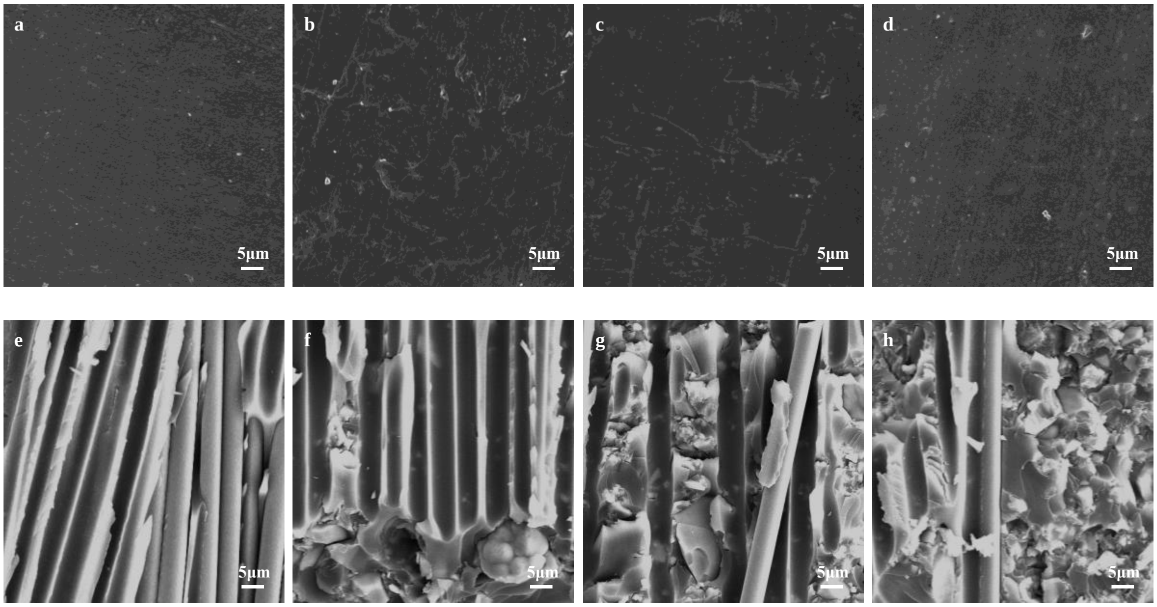

Figure S3. Scanning electron microscopy (SEM) images of the external surfaces of RTM epoxy composites preformed by the ET3228 tackifiers containing (a) 0, (b) 23, (c) 38, and (d) 48 wt % MRP, and fracture surfaces of RTM epoxy composites preformed by the ET3228 tackifiers containing (e) 0, (f) 23, (g) 38, and (h) 48 wt % MRP before flaming.

Table S1 MRP and ET3228 contents in composites

	COMP-T (0%P)	COMP-T (23%P)	COMP-T (38%P)	COMP-T (48%P)
MRP contents in composites (wt%)	0	0.83	1.66	2.5
ET3228 contents in composite (wt%)	2.67	2.67	2.67	2.67

Table S2 MRP and ET3228 contents in preforms

	COMP-T (0%P)	COMP-T (23%P)	COMP-T (38%P)	COMP-T (48%P)
MRP contents in preform (wt%)	0	1.25	2.5	3.75
ET3228 contents preform (wt%)	4	4	4	4

Table S3 First burning time t_1, secondary burning time t_2, and dripping observed for RTM composite samples preformed by the ET3228 tackifiers containing 0, 23, 38, and 48 wt% MRP, and cured resin samples containing 0, 2.5, 5, and 7.5 wt% MRP.

	t_1 (s)	t_2 (s)	Dripping	
COMP-T(0%P)	-	-	No	flammable
COMP-T(23%P)	25	5	No	self-extinguishing
COMP-T(38%P)	4	8	No	self-extinguishing
COMP-T(48%P)	2	2	No	self-extinguishing
Resin(0%P)	-	-	Yes	flammable
Resin(2.5%P)	-	-	Yes	flammable
Resin(5%P)	-	-	No	flammable
Resin(7.5%P)	-	-	No	flammable

Table S4 Combustion calorimetric test (CCT) results for the composites.

Composite	Peak of Heat Release Rate(kW/m^2)	Total Heat Release (MJ/m^2)	Residual weight (wt%)	Total smoke release (m^2/ m^2)
COMP-T (0%P)	234.24±5	46.82±1	66±0.1	2630±10
COMP-T (23%P)	191.33±5	33.07±1	69±0.1	3495±10
COMP-T (38%P)	199.34±5	32.73±1	68±0.1	3497±10
COMP-T (48%P)	211.87±5	32.66±1	68±0.1	3556±10
